# Validation of the Family Integration Experience Scale: Chronic Illness for the Portuguese language spoken in Brazil

**DOI:** 10.1590/1518-8345.6961.4248

**Published:** 2024-09-23

**Authors:** Carolliny Rossi de Faria Ichikawa, Regina Szylit

**Affiliations:** 1 Universidade de São Paulo, Escola de Enfermagem, Departamento Enfermagem Materno-Infantil e Psiquiátrica, São Paulo, SP, Brazil.; 2 Centro Universitário Faculdade de Medicina do ABC, Curso de Enfermagem, Santo André, SP, Brazil.

**Keywords:** Chronic disease, Family, Validation studies, Family relationships, Family nursing, Pediatric nursing

## Abstract

**Objective:**

to conduct the cross-cultural adaptation and psychometric validation of the Family Integration Experience Scale: Chronic Illness in Brazilian Portuguese.

**Method:**

a methodological study divided into two stages. In the first stage, the Family Integration Experience Scale: Chronic Illness was cross-culturally adapted for Brazilian culture, when the scale was subjected to translations, back-translations, and a committee of judges - to verify the semantic, linguistic, and contextual equivalence between the original and translated items. The second step was to validate the scale in a sample of families of children and adolescents with chronic illnesses. The participants were 230 families of children with chronic illnesses attending the outpatient clinic of a tertiary public hospital with teaching and research characteristics.

**Results:**

internal consistency was tested using Cronbach’s alpha (0.81) and McDonald’s omega (0.81). Confirmatory factor analysis was also tested, and the model’s fit was acceptable for validation standards.

**Conclusion:**

the version of the Family Integration Experience Scale: Chronic Illness showed evidence of validation and can be considered a valid and reliable instrument in Brazilian culture. The Brazilian Portuguese version of the Family Integration Experience Scale: Chronic Illness can be used to measure the experience of family integration in chronic illness.

## Highlights

**(1)** Validation of the Family Integration Experience Scale: Chronic Illness (FIES: CI).

**(2)** Family Integration Experience Scale: Chronic Illness.

**(3)** Optimal management of chronic illnesses involving interactions with health professionals.

**(4)** Strategies for strengthening family relationships in the context of chronic illness.

**(5)** Integration process used by family members to adapt to the illness.

## Introduction

 Scientific and technological innovations in neonatal and pediatric health care have led to significant changes in the epidemiology of chronic diseases in these phases of the life cycle. Infectious diseases have decreased substantially and life expectancy has increased, which has led to an increase in the prevalence of chronic diseases ^(^ 1 ^)^ . 

 Currently, chronic diseases represent the biggest cause of death and disability in the world, with around 74% of deaths attributed to them ^(^ 2 ^)^ . In Brazil, data indicate that between 9 and 11% of Brazilian children and adolescents suffer from chronic diseases - 9.1% of children aged zero to five, 9.7% of those aged six to thirteen, and 11% of adolescents aged fourteen to nineteen ^(^ 3 ^)^ . 

 The American Academy of Pediatrics (AAP) explains that a chronic health condition can last from three months to a lifetime, affecting the individual in their daily activities. In the United States of America (USA), between 10 and 20 million children and adolescents suffered from at least one chronic condition in 2020, out of a total of 72 million Americans under the age of 18 - this would add up to a percentage of children with chronic diseases of between 13 and 27% ^(^ 4 ^)^ . 

 In childhood, chronic diseases usually cause sequelae and limitations for the patient for a long time, having a major impact on many aspects of the child’s life and that of their family ^(^ 5 ^)^ . Optimal management of diseases and chronic health conditions involves adaptations in the daily routines of children and their families and in interactions with health professionals and services. 

 As families try to adapt to their child’s chronic illness, they make choices about their experiences and recognize their vulnerability and the current reality of chronic illness ^(^ 6 ^-^ 7 ^)^ . This ongoing process is called “family reintegration”; in it, families begin to use intentional care strategies and establish a commitment within the family with the aim of improving current and future family life ^(^ 7 ^)^ . 

 Family reintegration involves integration processes, which are used by family members to adapt to the reality and uncertainties of chronic illness, throughout the family life cycle in the context of chronic illness. Family reintegration enables family empowerment, helping them to adapt to reality and make choices within the experience of chronic illness ^(^ 6 ^-^ 7 ^)^ . Integration processes include recognizing the family and individual vulnerability of family members, intentional family care strategies, how to plan for monitoring and protection, and family engagement with chronic illness: how to connect, ponder, relate, and struggle ^(^ 7 ^)^ . 

 Mutual support between family members facilitates the adaptation process and the care of children with chronic illnesses. Families that share the decisions and care of the sick child have fewer difficulties in family life and in managing the disease, as well as less social vulnerability ^(^ 8 ^-^ 10 ^)^ . For the parents of a child with a chronic condition, emotional support among partners - family, friends, and health professionals - is essential for dealing with their child’s condition ^(^ 11 ^)^ . 

 Nurses are indispensable in helping these families find the best way to integrate. Faced with an illness, each family will act in a different way and this will depend on their previous experiences, their beliefs, the values of each family member, as well as the influence and space they develop and occupy in their organizational network ^(^ 12 ^)^ . In this experience, family members interact with each other and are influenced by each other, helping to develop, cope, maintain integrity, and build family health ^(^ 7 ^,^ 13 ^-^ 14 ^)^ . 

 Health professionals need to know about these experiences of family integration in the context of chronic illness so that they can develop family-centered approaches ^(^ 5 ^-^ 7 ^,^ 13 ^-^ 14 ^)^ . In order to provide care, it is necessary to understand the experiences of family integration in various chronic health conditions. However, the identification of family integration processes in Brazil is limited by the lack of valid and reliable measures that capture these experiences. 

 The Family Integration Experience Scale: Chronic Illness (FIES: CI) is a scale created in 2020 by American nurses, based on the theoretical model of Reintegration Within Families in the Context of Chronic Illness Model (RFCCI), with the aim of describing the family processes used to manage family life in the context of a chronic illness ^(^ 6 ^)^ . 

 The instrument addresses some central concepts, such as family vulnerability, family care strategies, family weighting, family relationships, family change, family connection, and family struggle ^(^ 6 ^-^ 7 ^)^ . It is made up of 9 items, presented in a visual-analog format, to capture the subjective nature of each process and increase measurement capacity ^(^ 6 ^)^ . 

 The scale was tested in the context of North American culture and proved to be reliable and applicable ^(^ 6 ^)^ . It is believed that the FIES: CI will be applicable and useful for Brazilian pediatric nursing, as it will provide a greater understanding of family integration and vulnerability in the face of chronic illness, making it possible to propose strategies for caring for and strengthening family relationships in the context of the child’s chronic illness. 

In Brazil, there is a lack of instruments or scales that describe family processes. As it is an easy-to-understand, self-administered scale, the Brazilian version of the FIES: CI could be used by pediatric nurses to get to know the families of children with chronic health conditions and to propose strategies to help these families in their daily lives with their children.

The aim of this study was to conduct the cross-cultural adaptation and psychometric validation of the Family Integration Experience Scale: Chronic Illness (FIES: CI) in Brazilian Portuguese.

## Method

### Study design

 This is a psychometric study of Cross-Cultural Adaptation (CCA) and validation of the FIES: CI instrument for the Portuguese language spoken in Brazil. The procedures recommended in the literature were followed ^(^ 15 ^)^ . This study was organized according to the recommendations of the Equator Network’s Revised Standards for Quality Improvement Reporting Excellence (SQUIRE 2.0) guide. 

### Setting

The study was divided into two phases, the first of which was to conduct the CCA of the FIES: CI scale for the Portuguese language spoken in Brazil, covering the following stages: translation; consensus of the translations; back-translation; expert committee; pre-finalization review; cognitive testing and linguistic validation and analysis of the comments.

The translation was performed by two translators fluent in English - the language of the original version. One translation was done by a health professional who knew the objectives of the study and the other by an English teacher who did not know the objectives of the study (blind translation), thus generating two versions. The two versions obtained from the initial translation stage were then reconciled to create the first version of the scale.

 Subsequently, the first reconciled version was sent to a native English translator, who back-translated the text into its original language ^(^ 15 ^-^ 16 ^)^ . This same reconciled version was sent to the expert committee, made up of health professionals - Brazilian and bilingual - with training and/or experience in family care, psychometrics, and linguistics. The role of the expert committee was to consolidate the final version of the instrument. 

The experts evaluated the translated version in terms of conceptual, cultural, semantic, and idiomatic equivalence. The experts were also asked to analyze items in order to validate the content of the instrument. The experts were contacted by e-mail.

In the second phase of the study, evidence of the validity and reliability of the Brazilian version of the scale was verified by means of construct validation. The instrument was first applied to five family members of children or adolescents with a chronic illness, to complete the pre-test. Cognitive testing and linguistic validation were then conducted. After the pre-test, the final version of the instrument was defined.

### Participants

 The psychometric properties of the scale were assessed in a cross-sectional study with 230 family members of children and adolescents aged up to 19 with chronic illnesses who were being monitored at the outpatient clinic of a public teaching hospital in the city of São Paulo. The sample was determined by convenience and exceeded what is recommended in the literature (10:1), allowing for more precise analysis ^(^ 17 ^-^ 18 ^)^ . The family member who took part in the survey had to be over 18 years old and live in the same household as the child or adolescent. 

### Period

The data was collected between March and May 2023.

### Instrument

 The FIES: CI is a self-administered scale and begins with an instructional introductory paragraph. The family member is instructed to mark the appropriate point along the visual-analog line that best describes the family members’ experiences of integrating the illness or chronic condition into family life. Next, the nine items are presented, in the format of a visual-analog scale score from 0 to 100. Three items are scored inversely for analysis: family care, family growth, and the importance of family relationships. The sum of the scores can be completed from 0 to 900, with lower scores indicating greater integration of the chronic illness into family life ^(^ 6 ^)^ . 

### Data collection

Participants were selected during the waiting period for their outpatient appointment and asked to complete a demographic characterization questionnaire and the final version of the FIES: CI.

Filling in the questionnaire took around 10 minutes and was done in the outpatient clinic, before or after the child’s/adolescent’s appointment. Complementary data, such as diagnosis and time of diagnosis, was collected from the patient’s medical records.

### Data analysis

 Construct validity is the extent to which a set of variables really represents the construct being measured. In this case, it was tested with Confirmatory Factor Analysis (CFA), using the Maximum Likelihood Method and the R statistical package, version 4.3.0. Items with a factor loading greater than 0.30 were acceptable ^(^ 17 ^,^ 19 ^)^ . 

 For the fit indices in the CFA, the following were tested: Comparative Fit Index (CFI) >0.90; Tucker-Lewis Index (TLI) >0.90; Root Mean Square Error of Approximation (RMSEA) <0.08) and Root Mean Square of Residuals (RMSR) <0.08 ^(^ 17 ^,^ 19 ^-^ 20 ^)^ . R-squared was calculated, which determines the proportion of variance in the dependent variable that can be explained by the independent variable; it represents the proportion of variability in the response variable explained by the predictor or explanatory variable. Known as the coefficient of determination: it gives an idea of how well the data fits the model ^(^ 21 ^)^ . 

 The reliability of the scale was tested by analyzing internal consistency, using Cronbach’s alpha (α) and McDonald’s Omega (ωt) coefficients, assessing the extent to which the parts of the scale measure the same characteristic - if their indices are worth up to 1. The higher the reliability, the more accurately the internal consistency is measured. For this study, a coefficient of 0.7 was adopted as satisfactory - an internationally accepted measure ^(^ 17 ^)^ . 

 The stability or reproducibility of the instrument (test-retest) was analyzed using the Intraclass Correlation Coefficient (ICC) ^(^ 17 ^,^ 22 ^)^ . 

The data was analyzed using the R statistical package, version 4.3.0. Descriptive statistics were used both to characterize the sample and to calculate frequency values, proportions, means, standard deviation, and minimum and maximum values. The significance level adopted for the tests was 5%.

### Ethical aspects

After authorization from the authors of the original scale, the project was submitted to the Ethics and Research Committee of the University of São Paulo’s College of Nursing and was granted Report No. 5.592,795. After clarifying the aim and procedures of the study, the participants signed the Informed Consent Form.

## Results

**Figure 1 -:**
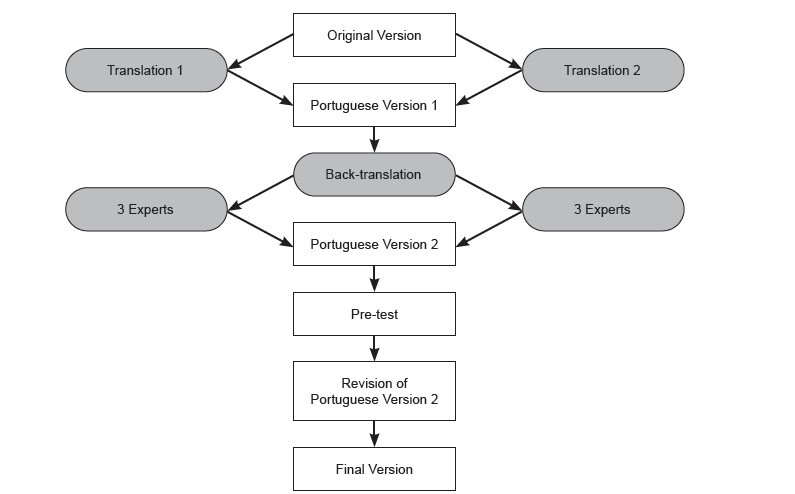
Stages of the FIES: CI cross-cultural adaptation process. São Paulo, SP, Brazil, 2023

**Figure 2 -:**
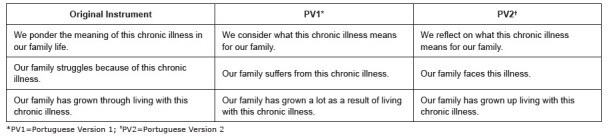
Items altered after evaluation by the expert committee. São Paulo, SP, Brazil, 2023

 Stage 1, considered to be the process of adapting the FIES: CI, included the translations, the synthesis of the translations, and the back-translation, as shown in Figure 1 . As there were no major differences in the two back-translated versions, Portuguese Version 1 (PV1) was produced. PV1 was sent to the expert committee for evaluation, with few suggestions for changes, as shown in Figure 2 , obtaining 80% agreement among the experts - making Portuguese Version 2 (PV2). 

In Stage 2, 230 family members of patients aged between 1 and 19 with chronic health conditions took part in the study, including mothers (78.2%), fathers (18.70%), grandmothers (2.17%) and aunts (0.87%). With regard to schooling: 21.30% had completed higher education; 1.74% had technical training; 56.09% had completed high school and 20.87% had completed elementary school.

Regarding the income of these families, 51.53% considered their family income to be sufficient, 45.85% considered their income to be insufficient and 2.62% reported having money left over at the end of the month. When asked about their income before the child’s illness, 65.7% of family members considered their family income to be sufficient; 19.65% considered their income to be insufficient and 15.28% reported having money left over at the end of the month.

Of the participating families, 162 had other children and 68 families had only one child: the child taking part in the study. The number of people living in the same household ranged from 2 to 8.

As for the children and adolescents, 132 (57.3%) were male and 98 (42.6%) female. Their ages ranged from 1 to 19 years.

The children’s diagnoses were distributed by system and were divided into neurological diseases (9.3); endocrine diseases; kidney diseases; respiratory diseases (13.4%); skin diseases (3.9%); liver diseases (4.3%); digestive system diseases (10%); genitourinary system diseases (2.61%); immune system diseases (6.09%); heart diseases (3.04%); genetic diseases (6.9); cancer (3.4%) and rare syndromes (14.3%).

As this is a two-dimensional model, the scale was tested on its two factors: Factor 1, which comprises items Q1, Q2, Q5, Q7, and Q9, which are considered negative factors; and Factor 2, with items: Q3, Q4, Q6, and Q8, considered positive factors.

A descriptive analysis of the performance of the scale items was performed. Cronbach’s alpha was used to measure internal reliability and was equal to 0.81, which means that 81% of the variability of the phenomenon can be explained by this scale. In the analysis of the alpha in the different factors of the scale, the reliability of Factor 1 was 0.81, while in Factor 2 it was 0.60.

*PV1=Portuguese Version 1

^†^ PV2=Portuguese Version 2

McDonald’s omega was also used as a reliability test, and a coefficient of 0.81 was obtained for the scale’s internal consistency, with a higher omega in Factor 1 (0.81) and Factor 2 (0.61).

 The characterization of each item of the scale individually was tested and showed that excluding items with low internal reliability values would not improve the value of Cronbach’s alpha in the factors, as shown in Table 1 . 

**Table 1:** - Descriptive statistics of the items that make up the scale. São Paulo, SP, Brazil, 2023

**Factor**	**Item**	n*	**Min** ^†^	**Max** ^‡^	**Mean**	**Standard Deviation**	**IC** ^§^	**Cronbach’s alpha Cdsi** ^||^	**McDonald’s Omega Cdsi** ^§^
**f1** ^¶^	1	230	0	100	48.63	39.52	0.582	0.781	0.782
	2	230	0	100	28.30	35.89	0.592	0.778	0.780
	5	230	0	100	31.16	37.29	0.613	0.772	0.773
	7	230	0	100	47.15	40.86	0.595	0.777	0.778
	9	230	0	100	38.30	41.21	0.621	0.769	0.769
f2**	3	230	0	100	80.30	30.21	0.349	0.557	0.560
	4	230	0	100	82.44	26.74	0.501	0.446	0.469
	6	230	0	100	84.15	29.21	0.304	0.590	0.595
	8	230	0	100	82.47	29.29	0.389	0.525	0.580

* n = Absolute number

† Min = Minimum

‡ Max = Maximum

§ IC = Item-Total Correlation

|| Cdsi = Coefficient of the domain without the item

¶ f1 = Factor 1

** f2 = Factor 2

 The correlation between the two factors of the scale was -0.637, with a 95% confidence interval of -0.864 to -0.409, as shown in Table 2 . 

The adjusted model showed adequate quality of fit indices between the scale items, but the correlation between its two factors was negative (-0.637). This was expected due to the inversion between the scale factors.

The RMSEA was 0.068 (90%CI: 0.044; 0.091), an adequate value and indicative of the model’s fit to the factor structure. The Standardized Root Mean Square Residuals (SRMR) value was 0.067 - also demonstrating the model’s fit to the factor structure. The incremental fit measures were TLI of 0.89 and CFI of 0.92. Thus, the structure of the instrument proved to be suitable for assessing the construct under investigation.

To assess the scale’s reliability, a test-retest was also performed. After four weeks, the scale was reapplied to 35 family members, obtaining an ICC value of 0.998 - showing a strong correlation.

**Table 2 -:** Distribution of factor loadings of the scale. São Paulo, SP, Brazil, 2023

Factor	Item	Factor load	Confidence interval	R-squared
**f** 1*	1	0.644	0.537 a 0.751	0.416
	2	0.715	0.575 a 0.854	0.513
	5	0.698	0.578 a 0.818	0.489
	7	0.653	0.544 a 0.763	0.429
	9	0.684	0.570 a 0.798	0.470
**f** 2 ^†^	3	0.654	0.424 a 0.883	0.429
	4	0.633	0.385 a 0.882	0.403
	6	0.350	0.103 a 0.596	0.123
	8	0.425	0.184 a 0.666	0.181

* f1 = Factor 1

† f2 = Factor 2w

## Discussion

The aim of this study was to conduct a cross-cultural adaptation and validate the FIESC: CI in Brazilian Portuguese. The FIESC: CI is a scale that aims to describe the experience of family integration in the context of chronic illness.

 The results of this study guarantee the validity and reliability of the Brazilian version of the FIES: CI. This is the first Brazilian instrument to measure the experience of family integration in the context of chronic illness. One of the strengths of the FIES: CI is that it is based on a solid conceptual framework, built up over decades of research, which makes it possible to assess and intervene in family integration in the context of chronic illness ^(^ 6 ^-^ 7 ^,^ 23 ^)^ . 

 In a CCA study, the semantic, linguistic, and contextual equivalence between the original and translated items should be assessed; the psychometric properties of the new version of the instrument should also be analyzed. The relevance of the concepts and domains covered by the original instrument in the new culture should also be assessed, as should the suitability of each item in the original instrument in terms of its ability to represent these concepts and domains in the target population ^(^ 24 ^)^ . 

 A systematic and rigorous method was used to translate and adapt the FIES: CI, which ensured the quality of the measure and provided evidence of the content validity, acceptability, and feasibility of the Brazilian version of the scale ^(^ 15 ^-^ 18 ^,^ 23 ^-^ 25 ^)^ . There were no major discrepancies in the versions presented by the translators, and those that did arise were promptly resolved by the study’s principal investigators. 

 In assessing the psychometric properties, the scale proved to be easy to apply and showed evidence of validity. This evidence was confirmed through factor analysis of the scale’s main components - the items that make up the factors indicate that the variance of these variables is reproduced by common factors ^(^ 19 ^-^ 20 ^)^ . 

 Factor analysis showed that all the items in the scale play an important role in describing the construct and that the factor loadings of the Brazilian version of the FIES: CI do not differ from the original version, maintaining the composition of the scale ^(^ 17 ^,^ 23 ^,^ 25 ^)^ . With the correlational analysis between the items of the scale and the global scale, it was concluded that the instrument can measure the family integration of children and adolescents with chronic conditions, as it shows that the correlations between all the items and the global scale are stronger than the correlations between the items alone ^(^ 17 ^,^ 19 ^)^ . 

 In the original instrument, factor analysis explained 63.8% of the variance, with a two-dimensional model with two factors: Factor 1, considered the negative integration factor, which evaluates family vulnerability; family changes; family suffering; family overload; and financial concerns; and Factor 2, the positive factor, which includes care strategies; the importance of family relationships and family reflection and growth ^(^ 6 ^)^ . 

 Supporting the construct validity of the Brazilian version, the internal consistency and test-retest reliability had excellent results, confirming that it is a reliable measure. The internal consistency value given by Cronbach’s alpha (0.81) exceeded the value obtained in the original instrument (0.79) ^(^ 6 ^)^ . The interpretation of the alpha coefficient is generally preferred over other interpretations; however, there is still no consensus in the literature about its interpretation ^(^ 26 ^)^ . 

 In this study, unlike the original study, previously published to develop and validate the FIES: CI scale ^(^ 6 ^)^ , reliability was also assessed using the McDonald’s omega coefficient, as it provides a more restrictive estimate of reliability ^(^ 26 ^)^ . 

 Considering test-retesting, a study to adapt and validate the Family Intensive Care Units Syndrome Inventory for Iranian culture emphasized its importance. The authors reapplied the instrument two weeks after its first application and the Intraclass Correlation Coefficient (ICC) found was 0.97 - a value similar to that found in this study ^(^ 22 ^,^ 27 ^)^ . The recommended time interval between the two measures is four weeks, to avoid variation in the perception of positive or negative symptoms and memory problems ^(^ 25 ^)^ . Changes in the child’s state of health that occur between tests can also influence respondents’ answers and reduce test-retest reliability. 

 Family competencies measured in the FIES: CI - such as care strategies, decision-making, and financial support - help develop problem-solving skills, reducing the chance of a negative outcome ^(^ 28 ^)^ . Satisfactory family relationships act as protective factors against risk behaviors for the family and its members ^(^ 28 ^-^ 29 ^)^ . 

 A Chinese study to build and validate an instrument that measures family regulation and integration confirmed that these are necessary. Children with families with excellent regulation and integration are 3.6 times more likely to be healthy than children with families that have difficulties in these two domains. The research also revealed that family conflict is associated with a 38% lower probability of being healthy ^(^ 13 ^)^ . 

 When validating the psychometric properties of the Family Health Scale instrument for the Brazilian version. The authors described the importance of considering all the factors in the scale - including measures such as family functioning, communication, problem-solving, coping, and emotional and economic support - as topics to be addressed when working with family health ^(^ 28 ^)^ . The authors also recommended that health professionals explore issues such as intra- and extra-family support bonds in their research, in order to enhance the understanding of health ^(^ 28 ^-^ 29 ^)^ . 

 Accurate, valid measuring instruments that address phenomena of interest in the fields of nursing and health are essential to aid professional practice and the development of research ^(^ 30 ^)^ . The FIES: CI Brazilian version was considered by the participants to be an easy-to-understand instrument - its good psychometric performance stems from this ease of completion. For a measuring instrument to be considered good, it needs to be well understood by its respondents, be pragmatic, reduce respondent burden, and capture the domains it sets out to measure ^(^ 31 ^)^ . 

The scale was named, in its Brazilian version, the Scale of Family Integration Experience in Chronic Illness, in an attempt to be as close as possible to the name of the original scale. It shows signs of reliability and validity and is ready to be used in Brazilian culture.

The instrument was administered in an outpatient clinic, during the wait for treatment. Many interviews were interrupted by medical appointments and had to be started again - which was a limiting factor in the study. There were also a lot of external stimuli, which distracted some participants.

Regarding its applicability, the participants did not find the instrument difficult to understand; however, in the first item (“Our family is feeling vulnerable with this chronic illness”), the word “vulnerability” was difficult to understand for a few participants, requiring an explanation of the concept of the word.

Collecting data in just one place was seen as a limitation, even though it was performed in a referral service, as most of the families lived in the metropolitan region. Knowing the cultural and socio-economic differences in our country, we believe it is important for the scale to be applied in other regions of Brazil.

It is also believed that this scale can be used as a tool to assess the experience of family integration in chronic illness, which will help health professionals establish better planning and development of care strategies for families.

The Family Integration Experience in Chronic Illness Scale can support the description and comparison of family members’ experiences in various chronic illness situations. In addition to simultaneously measuring experiences, it can represent the addition of a valuable tool to strategically inform family nursing therapeutic actions in the context of chronic illness.

## Conclusion

The Family Integration Experience Scale in Chronic Illness could contribute as an instrument to understand how families integrate in the various situations of chronic illness, with the stressful situations in their daily lives, knowing their vulnerability, and how these families strengthen themselves in the face of the various situations of illness.

The use of this tool in family assessment can provide greater interaction between health professionals and families, stimulating the planning of specific interventions for each family, and collaborating in the process of family integration in adapting to the illness. It also encourages families to seek strategies to strengthen family relationships in the context of the child’s chronic illness. It can be used in research in the field of nursing, making it possible to compare different realities - nationally and internationally.

The scale has been adapted and validated for families of children and adolescents with chronic illnesses. It presents sufficient evidence of content, construct, and criterion validity, in terms of the quality of the instrument, to describe the experience of family integration in a child’s chronic illness.

It is suggested that the sample should be expanded to include families of adults and the elderly with chronic conditions, to see how the instrument performs in these populations as well.
