# Supplementary material for: Validation of the Family Integration Experience Scale: Chronic Illness for the Portuguese language spoken in Brazil
Source: Rev Lat Am Enfermagem. 2024 Sep 23;32:e4248. doi: 10.1590/1518-8345.6961.4248 (PMC11421524; doi:10.1590/1518-8345.6961.4248)
Supplement: Translation

# Translation: Validación de la *Family Integration Experience Scale: Chronic Illness* para la Lengua Portuguesa hablada en Brasil

**DOI:** 10.1590/1518-8345.6961.4247

**Published:** 2024-09-23

**Authors:** Carolliny Rossi de Faria Ichikawa, Regina Szylit

**Affiliations:** 1 Universidade de São Paulo, Escola de Enfermagem, Departamento Enfermagem Materno-Infantil e Psiquiátrica, São Paulo, SP, Brasil.; 2 Centro Universitário Faculdade de Medicina do ABC, Curso de Enfermagem, Santo André, SP, Brasil.

**Keywords:** Enfermedad crónica, Familia, Estudios de validación, Relaciones familiares, Enfermería familiar, Enfermería pediátrica

## Abstract

**Objetivo:**

realizar la adaptación transcultural y la validación psicométrica de la *Family Integration* *Experience Scale: Chronic Illness* para la lengua portuguesa hablada en Brasil.

**Método:**

estudio metodológico, dividido en dos etapas. En la primera etapa, se realizó la adaptación transcultural de la *Family Integration Experience Scale: Chronic Illness* para la cultura brasileña; para esto, la escala fue sometida a traducciones, a retrotraducciones y a un comité de jueces, para verificación de la equivalencia semántica, lingüística y contextual entre los ítems originales y los traducidos. En la segunda etapa, se realizó la validación de la escala en una muestra de familias con niños y adolescentes con enfermedades crónicas. Los participantes fueron 230 familias de niños con enfermedades crónicas, atendidos en el ambulatorio de un hospital público terciario, con características de enseñanza e investigación.

**Resultados:**

la consistencia interna fue comprobada a través del alfa de Cronbach (0,81) y del Omega de McDonald (0,81). El análisis factorial confirmatorio también fue comprobado, habiéndose un ajuste del modelo de acuerdo con lo que es aceptable para los estándares de validación.

**Conclusión:**

la versión de la *Family Integration Experience Scale: Chronic Illness* presentó evidencias de validación y puede ser considerado un instrumento válido y confiable en la cultura brasileña. La *Family Integration Experience Scale: Chronic Illness* en la versión portuguesa de Brasil, puede ser utilizada para medir la experiencia de la integración familiar en la enfermedad crónica.

## Destacados

**(1)** Validación de la Family Integration Experience Scale: Chronic Illness (FIES:CI).

**(2)** Escala de experiencia de integración familiar frente a enfermedad crónica.

**(3)** Administración ideal de las enfermedades crónicas que contempla interacciones con profesionales de la salud.

**(4)** Estrategias de fortalecimiento de las relaciones familiares en el contexto de la enfermedad crónica.

**(5)** Proceso de integración usado por los miembros de la familia para adaptarse a la enfermedad.

## Introducción

Las innovaciones científicas y tecnológicas en la atención a la salud neonatal y pediátrica llevaron a importantes cambios en la epidemiología de las enfermedades crónicas, en las fases del ciclo vital. Las enfermedades infecciosas disminuyeron substancialmente y la expectativa de vida aumentó, reflejado en el aumento de la prevalencia de enfermedades crónicas ^(^ 1 ^)^ .

En la actualidad, las enfermedades crónicas representan la mayor causa de muerte y de incapacidades en el mundo; cerca de 74% de las muertes ocurridas son atribuidas a ellas ^(^ 2 ^)^ . En Brasil, en lo que refiere a la enfermedad crónica en el niño, los datos indican que de 9% a 11% de los niños y adolescentes brasileños son portadores de enfermedad crónica, distribuidos en 9,1% de niños de cero a cinco años, 9,7% de seis a trece años y 11% de adolescentes entre catorce y diecinueve años ^(^ 3 ^)^ .

 La *American Academy of Pediatrics* (AAP) esclarece que una condición crónica de salud puede durar de tres meses hasta toda la vida, afectando al individuo en sus actividades diarias. En Estados Unidos (EUA), entre 10 y 20 millones de niños y adolescentes sufrían de, por lo menos, una condición crónica en 2020 y, un total de 72 millones de norteamericanos menores de 18 años, lo que resultaría en un porcentaje de niños con enfermedades crónicas, entre 13% y 27% ^(^ 4 ^)^ . 

En la infancia las enfermedades crónicas, generalmente, causan secuelas y limitaciones al paciente por un largo tiempo, lo que provoca un gran impacto en muchos aspectos de la vida del niño y de su familia ^(^ 5 ^)^ . La administración ideal de enfermedades y condiciones crónicas de salud comprende adaptaciones en las rutinas diarias de los niños y sus familias y en las interacciones con profesionales y servicios de salud.

A medida que las familias tratan de adaptarse a la enfermedad crónica del niño, hacen elecciones relacionadas con sus experiencias y reconocen su vulnerabilidad y la actual realidad de la enfermedad crónica ^(^ 6 ^-^ 7 ^)^ . Ese proceso continuo es denominado “reintegración familiar”; en este, las familias pasan a utilizar estrategias intencionales de cuidado y establecen un compromiso dentro de la familia, con el objetivo de mejorar la vida familiar actual y futura ^(^ 7 ^)^ .

La reintegración familiar está relacionada con procesos de integración, usados por los miembros de la familia para adaptarse a la realidad y a las incertezas de la enfermedad crónica, a lo largo del ciclo de vida familiar, en el contexto de la enfermedad crónica. La reintegración familiar posibilita la capacitación de la familia, auxiliándola a adaptarse a la realidad y a hacer elecciones dentro de la experiencia de la enfermedad crónica ^(^ 6 ^-^ 7 ^)^ . Los procesos de integración incluyen: el reconocimiento de la vulnerabilidad familiar e individual de los miembros de la familia; las estrategias intencionales de cuidado familiar, como planificar el monitoreo; la protección; y, el compromiso familiar con la enfermedad crónica, como conectarse, ponderar, relacionarse y luchar ^(^ 7 ^)^ .

El apoyo mutuo entre los familiares se torna un facilitador para el proceso de adaptación y para los cuidados de niños con enfermedades crónicas. Las familias que comparten las decisiones y los cuidados del niño enfermo tiene menos dificultades en la vida familiar y en el control de la enfermedad, así como menor vulnerabilidad social ^(^ 8 ^-^ 10 ^)^ . Para los padres del niño con condición crónica, es fundamental el apoyo emocional entre los compañeros (familia, amigos y profesionales de la salud) para lidiar con la condición del hijo ^(^ 11 ^)^ .

Los enfermeros son indispensables para auxiliar a las familias a encontrar la mejor forma de integración. Delante de una situación de enfermedad, cada familia actuará de una manera diferente y eso depende de sus experiencias previas, creencias y valores de cada miembro familiar, que está vinculado a la influencia y al espacio que desarrollan y ocupan en su red organizacional ^(^ 12 ^)^ . Al utilizar la experiencia, los miembros de la familia interactúan entre sí, influenciándose entre ellos, auxiliando el desarrollo, el enfrentamiento, la manutención de la integridad y la construcción de la salud familiar ^(^ 7 ^,^ 13 ^-^ 14 ^)^ .

Los profesionales de la salud deben conocer esas experiencias de integración familiar en el contexto de la enfermedad crónica, para que puedan elaborar abordajes centrados en la familia ^(^ 5 ^-^ 7 ^,^ 13 ^-^ 14 ^)^ . Para atender es necesario comprender las experiencias de integración familiar, en las diversas condiciones crónicas de salud. Sin embargo, la identificación de los procesos de integración familiar, en la realidad brasileña, es limitada por la falta de mediciones válidas y confiables que capturen esas experiencias.

 La *Family Integration Experience Scale: Chronic Illness* (FIES:CI) es una escala creada en 2020, por enfermeras norteamericanas, a partir del modelo teórico *Reintegration Within* *Families in the Context of Chronic Illness Model* (RFCCI), con el objetivo de describir los procesos familiares utilizados para administrar la vida familiar en el contexto de una enfermedad crónica ^(^ 6 ^)^ . 

El instrumento aborda algunos conceptos centrales, como: vulnerabilidad familiar, estrategias de cuidado familiar, ponderación familiar, relacionamiento familiar, transformación familiar, conexión familiar y lucha familiar ^(^ 6 ^-^ 7 ^)^ . Está compuesto por 9 ítems, presentados en formato analógico-visual, para capturar lo subjetivo de cada proceso y aumentar la capacidad de medición ^(^ 6 ^)^ .

La escala fue comprobada en el contexto de la cultura norteamericana y se mostró confiable y aplicable ^(^ 6 ^)^ . Se cree que la FIES:CI será aplicable y útil para la enfermería pediátrica brasileña, por proporcionar mayor comprensión de la integración y de la vulnerabilidad familiar, delante de la enfermedad crónica, posibilitando proponer estrategias de cuidado y fortalecimiento de las relaciones familiares, en el contexto de la enfermedad crónica del niño.

En la realidad brasileña, existe un vacío en lo que se refiere a instrumentos o escalas que describen procesos familiares. Por tratarse de una escala de fácil comprensión y ser autoaplicable, la versión brasileña de la FIES:CI podrá ser utilizada, por la enfermería pediátrica, para conocer a las familias con niños en condiciones crónicas de salud y para permitir proponer estrategias para auxiliar a esas familias en el día a día con sus niños.

 El objetivo de este estudio fue realizar la adaptación transcultural y la validación psicométrica de la *Family Integration Experience Scale: Chronic Illness* (FIES:CI) para la lengua portuguesa hablada en Brasil. 

## Método

### Delineamiento del estudio

 Se trata de un estudio psicométrico de Adaptación Transcultural (ATC) y validación del instrumento FIES:CI para la lengua portuguesa hablada en Brasil. Para esto, fueron seguidos los procedimientos recomendados en la literatura ^(^ 15 ^)^ . Este estudio fue organizado de acuerdo con las recomendaciones de la guía *Revised Standards for Quality Improvement Reporting Excellence* (SQUIRE 2.0), de la *Rede Equator* . 

### Escenario

El estudio fue dividido en dos fases, siendo la primera la realización del ATC de la escala FIES:CI para la lengua portuguesa hablada en Brasil, conteniendo las etapas: traducción; consenso de las traducciones; retrotraducción; comité de especialistas; revisión de pre-finalización; test cognitivo; validación lingüística; y, análisis de los comentarios.

La traducción fue realizada por dos traductores fluentes en el idioma inglés, que es el idioma de la versión original. Una traducción fue realizada por un profesional del área de la salud que conocía los objetivos del estudio y la otra por un profesor de inglés que no conocía los objetivos del estudio (traducción ciega), generándose así dos versiones. Después, se realizó una conciliación de las dos versiones obtenidas en la etapa inicial de la traducción, elaborándose así la primera versión de la escala.

Posteriormente, la primera versión conciliada fue enviada para un traductor nativo de la lengua inglesa, que realizó la retrotraducción, es decir, la vuelta del texto para el idioma de origen ^(^ 15 ^-^ 16 ^)^ . Esa misma versión conciliada fue enviada para el comité de especialistas, compuesto por profesionales de la salud (brasileños y bilingües) con formación y/o experiencia en cuidado familiar, psicometría y lingüística. El papel del comité de especialistas fue consolidar la versión final del instrumento.

Los especialistas evaluaron la versión traducida considerando el análisis de las equivalencias conceptuales, culturales, semánticas e idiomáticas. También se solicitó a los especialistas el análisis de los ítems con el objetivo de validar el contenido del instrumento. El contacto con los especialistas fue a través del correo electrónico.

En la segunda fase del estudio, fue realizada la verificación de evidencias de validez y confiabilidad de la versión brasileña de la escala, por medio de la validación de constructo. El instrumento fue aplicado inicialmente en cinco familiares con niños o adolescentes con enfermedad crónica, para la realización del pretest. Posteriormente, se realizó el test cognitivo y la validación lingüística. Después de la aplicación del pretest, fue definida la versión final del instrumento.

### Participantes

La evaluación de las propiedades psicométricas de la escala fue realizada a través de un estudio transversal, con 230 familiares de niños y adolescentes, con hasta 19 años y con enfermedades crónicas, que estaban siendo acompañados en el ambulatorio de un hospital-escuela público en la ciudad de Sao Paulo. La determinación de la muestra fue realizada por conveniencia y superó lo recomendado por la literatura (de 10:1), posibilitando análisis más precisos ^(^ 17 ^-^ 18 ^)^ . El familiar que respondería a la investigación debería tener más de 18 años y vivir en la misma residencia que el niño o adolescente.

### Período

La recolección de datos fue realizada entre los meses de marzo y mayo de 2023.

### Instrumento

La FIES:CI es una escala autoaplicable y se inicia con un parágrafo introductorio de instrucciones, en el cual el miembro de la familia es instruido a marcar el punto apropiado a lo largo de la línea analógica-visual, que mejor describa las experiencias de los miembros de la familia con la integración de la enfermedad o la condición crónica en la vida familiar. En seguida, los nueve ítems son presentados, en el formato de puntuación de escala analógica-visual de 0 a 100. Tres ítems son puntuados inversamente para el análisis: cuidado familiar, crecimiento familiar e importancia del relacionamiento familiar. La suma de los puntajes puede completar de 0 a 900, siendo que puntajes más bajos indican mayor integración de la enfermedad crónica en la vida familiar ^(^ 6 ^)^ .

### Recolección de los datos

Los participantes fueron seleccionados durante el período de espera en la consulta de ambulatorio, siendo convidados a responder a un cuestionario de caracterización demográfica y a la versión final del FIES:CI.

El llenado del instrumento tomó cerca de 10 minutos y fue realizado en el ambulatorio, antes o después de la consulta del niño o adolescente. Los datos complementarios, como diagnóstico y tiempo de diagnóstico, fueron recolectados en la ficha médica del paciente.

### Análisis de los datos

 La validez de constructo es la extensión en que un conjunto de variables, realmente, representa el constructo a ser medido. En este estudio, la validez fue comprobada con el Análisis Factorial Confirmatorio (AFC), utilizando el Método de la Máxima Verosimilitud y con el *software* estadístico R, versión 4.3.0. Fueron aceptables los ítems con carga factorial mayor que 0,30 ^(^ 17 ^,^ 19 ^)^ . 

 Para los índices de ajustamiento, en la AFC, fueron comprobados con: *Comparative Fit Index* (CFI) >0,90; *Tucker-Lewis Index* (TLI) >0,90; *Root Mean Square Error of Aproximation* (RMSEA) <0,08) y *Root Mean Square of Residuals* (RMSR) <0,08 ^(^ 17 ^,^ 19 ^-^ 20 ^)^ . Fue calculado el R-cuadrado, que determina la proporción de variancia en la variable dependiente, que puede ser explicada por la variable independiente; este representa la proporción de la variabilidad en la variable respuesta explicada por la variable predictora o por la variable explicativa. Conocido como coeficiente de determinación, suministra una idea de cuán bien los datos se ajustan al modelo ^(^ 21 ^)^ . 

La confiabilidad de la escala fue comprobada por medio del análisis de la consistencia interna, utilizando los coeficientes de alfa de Cronbach (α) y Omega de McDonald (ωt), evaluando hasta qué punto las partes de la escala miden la misma característica; el índice máximo vale 1. Cuanto más alta es la confiabilidad, más exacta será la medida de la consistencia interna. Para este estudio, se adoptó un coeficiente de 0,7 como satisfactorio, valor aceptado y considerado internacionalmente ^(^ 17 ^)^ .

La estabilidad o reproductibilidad del instrumento (test-retest) fue analizada por el Coeficiente de Correlación Intraclase (ICC) ^(^ 17 ^,^ 22 ^)^ .

 Los datos fueron analizados por medio del *software* estadístico R, versión 4.3.0 *.* Se utilizó la estadística descriptiva, tanto para caracterización de la muestra como para el cálculo de los valores de frecuencia, proporciones, medias, desviación estándar y valores mínimos y máximos. El nivel de significación adoptado en las pruebas fue de 5%. 

### Aspectos éticos

Después de la autorización de las autoras de la escala original, el proyecto fue sometido al Comité de Ética e Investigación de la Escuela de Enfermería de la Universidad de São Paulo, obteniendo la Decisión nº 5.592.795. Después de los esclarecimientos sobre el objetivo y los procedimientos del estudio, los participantes firmaron el Término de Consentimiento Libre e Informado.

## Resultados

 La Etapa 1 considerada como el proceso de adaptación del FIES:CI, comprendió las traducciones, la síntesis de las traducciones y la retrotraducción, conforme se presenta en la Figura 1 . No habiéndose encontrado grandes divergencias en las dos versiones retrotraducidas, fue elaborada la Versión Portugués 1 (VP1). La VP1 fue encaminada para la evaluación del comité de especialistas, con pocas sugestiones de alteraciones, conforme demostrado en la Figura 2 , obteniéndose 80% de concordancia entre los especialistas; información utilizada para la construcción de la Versión Portugués 2 (VP2). 

En la Etapa 2, participaron del estudio 230 familiares de pacientes de 1 a 19 años, con condiciones crónicas de salud, incluyéndose: madre (78,2%), padre (18,70%), abuela (2,17%) y tía (0,87%). En relación a la escolaridad: 21,30% concluyeron el nivel superior; 1,74% tenían formación técnica; 56,09% tenían enseñanza media completa; y, 20,87% habían concluido la enseñanza fundamental.

En relación a la renta de esas familias, 51,53% consideraron la renta familiar suficiente, 45,85% consideraron tener renta insuficiente y 2,62% relataron sobrar dinero al final del mes. Al ser cuestionados en relación a la renta antes de la enfermedad del niño, 65,7% de los familiares consideraron que la renta familiar era suficiente; 19,65% consideraron la renta insuficiente y 15,28% relataron que sobraba dinero al final del mes.

**Figura 1 -:** Etapas del proceso de adaptación transcultural de la FIES:CI. São Paulo, SP, Brasil, 2023

*VP1=Versión Portugués 1; VP2=Versión Portugués 2

**Figura 2 -:** Ítems alterados después de la evaluación del comité de especialistas. São Paulo, SP, Brasil, 2023

Entre las familias de los participantes, 162 familias tenían otros hijos y 68 solamente un hijo, participante del estudio. La cantidad de personas que residían en la misma residencia varió de 2 a 8 personas.

En cuanto a los niños y adolescentes, 132 (57,3%) eran del sexo masculino y 98 (42,6%) del sexo femenino. La edad varió de 1 a 19 años.

Los diagnósticos de los niños fueron distribuidos por los sistemas, siendo divididos en enfermedades: neurológicas (9,3); endócrinas; renales; respiratorias (13,4%); de piel (3,9%); hepáticas (4,3%); del sistema de digestión (10%); del sistema genitourinario (2,61%); del sistema inmunológico (6,09%); cardíacas (3,04%); genéticas (6,9); de cáncer (3,4%); y, de síndromes raros (14,3%).

Como se trata de un modelo bidimensional, la escala fue comprobada en sus dos factores: Factor 1, que comprende los ítems Q1, Q2, Q5, Q7 y Q9, siendo considerados factores negativos; y Factor 2, con los ítems: Q3, Q4, Q6 y Q8, considerados factores positivos.

Se realizó el análisis descriptivo del desempeño de los ítems de la escala. El alfa de Cronbach fue usado para medir la confiabilidad interna, siendo igual a 0,81, que permitió afirmar que 81% de la variabilidad del fenómeno puede ser explicado por esa escala. En el análisis del alfa, en los diferentes factores de la escala, la confiabilidad del Factor 1 fue de 0,81, en cuanto en el Factor 2 fue de 0,60.

Fue realizado también, como test de confiabilidad el Omega de McDonald, obteniéndose un coeficiente de 0,81 para la consistencia interna de la escala, presentando un Omega de mayor valor en el Factor 1 (0,81) y en el Factor 2 (0,61).

 La caracterización de cada ítem de la escala, individualmente, fue comprobada y demostró que la exclusión de ítems con bajo valor de confiabilidad interna no mejoraría el valor del alfa de Cronbach en los factores, conforme se presenta en la Tabla 1 . 

 La correlación entre los dos factores de la escala fue de -0,637, con intervalo de confianza de 95% -0,864 a-0,409, conforme se presenta en la Tabla 2 . 

El modelo ajustado presentó adecuados índices de calidad de ajustamiento, entre los ítems de la escala; sin embargo, la correlación entre sus dos factores fue negativa (-0,637), hecho que era esperado, debido a la inversión entre los factores de la escala.

**Tabla 1 -:** Estadística descriptiva de los ítems que componen la escala. São Paulo, SP, Brasil, 2023

**Factor**	**Ítem**	n*	**Mín**	**Máx**	**Media**	**Desviación Estándar**	**CI**	**Alfa de Cronbach Cdsi**	**Omega de McDonald Cdsi**
**f** 1	1	230	0	100	48,63	39,52	0,582	0,781	0,782
	2	230	0	100	28,30	35,89	0,592	0,778	0,780
	5	230	0	100	31,16	37,29	0,613	0,772	0,773
	7	230	0	100	47,15	40,86	0,595	0,777	0,778
	9	230	0	100	38,30	41,21	0,621	0,769	0,769
**f** 2**	3	230	0	100	80,30	30,21	0,349	0,557	0,560
	4	230	0	100	82,44	26,74	0,501	0,446	0,469
	6	230	0	100	84,15	29,21	0,304	0,590	0,595
	8	230	0	100	82,47	29,29	0,389	0,525	0,580

* n = Número absoluto; Mín = Mínimo; Max = Máximo; CI = Correlación Ítem-Total; Cdsi = Coeficiente del dominio sin el ítem; f1 = Factor 1

** f2 = Factor 2

**Tabla 2 -:** Distribución de las cargas factoriales de la escala. São Paulo, SP, Brasil, 2023

Factor	Ítem	Carga factorial	Intervalo de confianza	R-cuadrado
**f**1*	1	0,644	0,537 a 0,751	0,416
	2	0,715	0,575 a 0,854	0,513
	5	0,698	0,578 a 0,818	0,489
	7	0,653	0,544 a 0,763	0,429
	9	0,684	0,570 a 0,798	0,470
**f2**	3	0,654	0,424 a 0,883	0,429
	4	0,633	0,385 a 0,882	0,403
	6	0,350	0,103 a 0,596	0,123
	8	0,425	0,184 a 0,666	0,181

* f1 = Factor 1; f2 = Factor 2

 La RMSEA fue de 0,068 (IC90%: 0,044; 0,091), valor adecuado e indicativo de ajuste del modelo a la estructura factorial. El valor del *Standardized Root Mean Square Residuals* (SRMR) fue de 0,067, también demostrando ajuste del modelo a la estructura factorial. Las medidas de ajuste incrementales fueron TLI de 0,89 e CFI de 0,92. Así, la estructura del instrumento se mostró adecuada para evaluación del constructo investigado. 

Para evaluar la confiabilidad de la escala, fue realizado también el test-retest. Después de cuatro semanas, la escala fue nuevamente aplicada a 35 familiares, obteniéndose un valor de ICC de 0,998, que demuestra fuerte correlación.

## Discusión

La presente investigación tuvo como objetivos realizar la adaptación transcultural y validar la FIES:CI para la lengua portuguesa hablada en Brasil. La FIESC:CI es una escala que se propone describir la experiencia de la integración familiar, en el contexto de la enfermedad crónica.

Los resultados de este estudio garantizan la validez y confiabilidad de la versión brasileña de la FIES:CI. Este es el primer instrumento brasileño que objetiva medir la experiencia de la integración familiar, en el contexto de la enfermedad crónica. Un punto fuerte de la FIES:CI es que está basada en una estructura conceptual sólida, construida a través de décadas de investigación, que permite evaluar e intervenir en la integración familiar, en el contexto de la enfermedad crónica ^(^ 6 ^-^ 7 ^,^ 23 ^)^ .

En una investigación de ATC, se debe evaluar las equivalencias semántica, lingüística y contextual, entre los ítems originales y los traducidos; también debe ser realizado el análisis de las propiedades psicométricas de la nueva versión del instrumento. La pertinencia de los conceptos y de los dominios contenidos en el instrumento original en la nueva cultura, también debe ser evaluada; así como, es necesario considerar la adecuación de cada ítem del instrumento original, en términos de la capacidad de representar esos conceptos y dominios, en la población objeto de la investigación ^(^ 24 ^)^ .

En la traducción y adaptación de la FIES:CI, se utilizó un método sistemático y riguroso, que garantizó la calidad de la medida y suministró evidencias de la validez, de la aceptabilidad y de la viabilidad del contenido de la escala en su versión brasileña ^(^ 15 ^-^ 18 ^,^ 23 ^-^ 25 ^)^ . No hubo grandes discrepancias entre las versiones presentadas por los traductores; aquellas que surgieron fueron rápidamente solucionadas por las investigadoras principales del estudio.

En la evaluación de las propiedades psicométricas, la escala se mostró fácil de ser aplicada, demostrando evidencias de su validez, las que fueron confirmadas a través del análisis factorial de los principales componentes de la escala. Los ítems que componen los factores indican que la variancia de esas variables es reproducida por factores comunes ^(^ 19 ^-^ 20 ^)^ .

Con el análisis factorial fue verificado que todos los ítems de la escala tienen un papel importante en la descripción del constructo y que las cargas factoriales, de la versión brasileña de la FIES:CI, no divergen de la versión original; así, manteniéndose la composición de la escala ^(^ 17 ^,^ 23 ^,^ 25 ^)^ . Con el análisis correlacional entre los ítems de la escala y la escala global, se concluyó que el instrumento puede medir la integración familiar de niños y adolescentes con condiciones crónicas, ya que muestra que las correlaciones entre todos los ítems y la escala global son más fuertes que las correlaciones apenas entre los ítems ^(^ 17 ^,^ 19 ^)^ .

En el instrumento original, el análisis factorial explicó 63,8% de la variancia, con un modelo bidimensional con dos factores. El Factor 1 considerado como factor de integración negativo evalúa: la vulnerabilidad familiar; las transformaciones de la familia; el sufrimiento de la familia; la sobrecarga de la familia; y, las preocupaciones financieras. El Factor 2, considerado factor positivo incluye: estrategias de cuidado; importancia de las relaciones familiares; y, reflexión y crecimiento de la familia ^(^ 6 ^)^ .

Apoyando la validez del constructo de la versión brasileña, la consistencia interna y la confiabilidad test-retest tuvieron resultados excelentes, confirmando que se trata de una medida confiable. El valor de la consistencia interna verificada con el alfa de Cronbach (0,81) superó el valor obtenido en el instrumento original (0,79) ^(^ 6 ^)^ . La interpretación del coeficiente alfa es generalmente preferida en detrimento de otras interpretaciones; sin embargo, todavía no existe consenso en la literatura acerca de su interpretación ^(^ 26 ^)^ .

En este estudio, diferentemente del estudio original publicado anteriormente, para desarrollar y validar la escala FIES:CI ^(^ 6 ^)^ , la confiabilidad también fue evaluada utilizando el coeficiente Omega de McDonald’s, que suministra una estimativa más restrictiva de la confiabilidad ^(^ 26 ^)^ .

 Considerando la realización del test-retest, un estudio para adaptar y validar la *Family* *Intensive Care Units Syndrome Inventory* , en la cultura de Irán, enfatizó la importancia de su realización. Los autores aplicaron nuevamente el instrumento dos semanas después de su primera aplicación; el Coeficiente de Correlación Intraclase (ICC) encontrado fue de 0,97, valor similar al encontrado en este estudio ^(^ 22 ^,^ 27 ^)^ . Para evitar la variación de la percepción de los síntomas positivos o negativos y los problemas de memoria, el intervalo de tiempo recomendado entre las dos medidas es de cuatro semanas ^(^ 25 ^)^ . Los cambios en el estado de salud del niño, que ocurren entre las pruebas, también pueden influenciar las respuestas de los entrevistados, lo que disminuye la confiabilidad del test-retest. 

Las competencias familiares, medidas en la FIES:CI, como las estrategias para el cuidado, la toma de decisiones y el apoyo financiero, auxilian a desarrollar competencias dirigidas a la resolución de problemas, reduciendo la chance de un resultado negativo ^(^ 28 ^)^ . Las relaciones familiares satisfactorias funcionan como factores de protección contra los comportamientos de riesgo para los miembros de la familia ^(^ 28 ^-^ 29 ^)^ .

Un estudio realizado en China, para construcción y validación de un instrumento que mide la regulación y la integración familiar, confirmó que esos dominios son necesarios, ya que los niños con familias con excelente regulación e integración tienen 3,6 veces más probabilidad de ser saludables, si comparados con niños con familias con dificultades en esos dos dominios. La investigación también reveló que el conflicto familiar está asociado a una probabilidad 38% menor de ser saludables ^(^ 13 ^)^ .

 Al validar las propiedades psicométricas del instrumento *Family Health Scale* en la versión brasileña, los autores describieron la importancia de considerar todos los factores de la escala; entre ellos medidas como: funcionamiento familiar, comunicación, resolución de problemas, *coping* , soporte emocional y económico; temas que deben ser abordados al trabajar con la salud de la familia ^(^ 28 ^)^ . Además, los autores recomendaron que los profesionales de la salud exploren, en sus investigaciones, cuestiones como los vínculos de apoyo intra y extrafamiliar, con la finalidad de potencializar la comprensión sobre la salud ^(^ 28 ^-^ 29 ^)^ . 

Instrumentos de medición precisos y válidos que abordan fenómenos de interés de las áreas de la Enfermería y de la Salud, son fundamentales para auxiliar la práctica profesional y el desarrollo de investigaciones ^(^ 30 ^)^ . La versión brasileña de la FIES:CI fue considerada por los participantes un instrumento de fácil comprensión; su buen desempeño psicométrico proviene de su facilidad de llenado. Para que un instrumento de medida sea considerado bueno, necesita: ser bien comprendido por los encuestados; ser pragmático; reducir la sobrecarga del encuestado; y, capturar los dominios que se propone medir ^(^ 31 ^)^ .

La escala fue denominada, en su versión brasileña, como Escala de Experiencia de Integración Familiar en la Enfermedad Crónica, buscando la máxima proximidad con la denominación de la escala original; esta presenta indicios de confiabilidad y validez y se encuentra disponible para ser utilizada en la cultura brasileña.

La aplicación del instrumento fue realizada en un ambulatorio, durante la espera para la atención médica. Muchas entrevistas fueron interrumpidas por las consultas médicas, precisando ser iniciadas nuevamente, esto fue un factor limitante del estudio. También, hubo muchos estímulos externos, que provocaron la distracción en algunos participantes.

En relación a su aplicabilidad, los participantes no consideraron el instrumento difícil de ser comprendido; sin embargo, en el primer ítem (“Nuestra familia está sintiéndose vulnerable con esta enfermedad crónica”), la palabra “vulnerabilidad” representó la dificultad de comprensión para pocos participantes, siendo necesaria ofrecer una explicación sobre el concepto de esa palabra.

La recolección de datos, en apenas un local, fue entendida como una limitación, a pesar de ser realizada en un servicio de referencia, ya que las familias, en su mayoría, residían en la región metropolitana. Conociendo las diferencias culturales y socioeconómicas de nuestro país, pensamos que es importante que la escala sea aplicada en otras regiones de Brasil.

También, pensamos que esa escala podrá ser utilizada como herramienta para evaluación de la experiencia de la integración familiar, en la enfermedad crónica, lo que auxiliará a los profesionales de la salud a mejorar la planificación y el desarrollo de estrategias de atención y cuidado de las familias.

La Escala de Experiencia de Integración Familiar en la Enfermedad Crónica puede apoyar la descripción y comparación de las experiencias de los miembros de la familia, en varias situaciones de enfermedades crónicas. Además de medir simultáneamente las experiencias, la escala puede representar la adición de una herramienta valiosa para informar estratégicamente las acciones terapéuticas de enfermería familiar, en el contexto de la enfermedad crónica.

## Conclusión

La Escala de Experiencia de Integración Familiar en la Enfermedad Crónica podrá contribuir como un instrumento para comprender cómo las familias se integran en diversas situaciones de la enfermedad crónica y en situaciones estresantes, en su cotidiano; al conocer sus vulnerabilidades esas familias se fortalecen delante de las diversas situaciones de la enfermedad.

La utilización de este instrumento en la evaluación familiar puede proporcionar mayor interacción de los profesionales de la salud con las familias, estimulando la planificación de intervenciones específicas para cada familia, colaborando con el proceso de integración familiar en la adaptación a la enfermedad e incentivando las familias a buscar estrategias de fortalecimiento de las relaciones familiares, en el contexto de la enfermedad crónica del niño. También, podrá ser utilizado en la investigación, en el campo de la Enfermería, permitiendo la comparación entre diversas realidades nacionales e internacionales.

La escala fue adaptada y validada en familias de niños y adolescentes con enfermedades crónicas. Ella presenta evidencias de validez de contenido, constructo y criterios suficientes, en lo que se refiere a la calidad del instrumento para describir la experiencia de la integración familiar, en la enfermedad crónica del niño.

Se sugiere la ampliación de la muestra para familias de adultos y adultos mayores con condiciones crónicas, para verificar cómo el instrumento se comporta en esas poblaciones.

# Translation: Validação da *Family Integration Experience Scale: Chronic Illness* para a Língua Portuguesa falada no Brasil

**DOI:** 10.1590/1518-8345.6961.4249

**Published:** 2024-09-23

**Authors:** Carolliny Rossi de Faria Ichikawa, Regina Szylit

**Affiliations:** 1 Universidade de São Paulo, Escola de Enfermagem, Departamento Enfermagem Materno-Infantil e Psiquiátrica, São Paulo, SP, Brasil.; 2 Centro Universitário Faculdade de Medicina do ABC, Curso de Enfermagem, Santo André, SP, Brasil.

**Keywords:** Doença crônica, Família, Estudos de validação, Relações familiares, Enfermagem familiar, Enfermagem pediátrica

## Abstract

**Objetivo:**

realizar a adaptação transcultural e a validação psicométrica da *Family Integration* *Experience Scale: Chronic Illness* para a língua portuguesa falada no Brasil.

**Método:**

estudo metodológico, dividido em duas etapas. Na primeira etapa, realizou-se a adaptação transcultural da *Family Integration Experience Scale: Chronic Illness* para a cultura brasileira, quando a escala foi submetida a traduções, a retrotraduções e a um comitê de juízes – para verificação da equivalência semântica, linguística e contextual entre os itens originais e traduzidos. Na segunda, realizou-se a validação da escala em amostra de famílias de crianças e adolescentes com doenças crônicas. Os participantes foram 230 famílias de crianças com doenças crônicas em atendimento no ambulatório de um hospital público terciário, com características de ensino e pesquisa.

**Resultados:**

a consistência interna foi testada por meio do alfa de Cronbach (0,81) e do ômega de McDonald (0,81). A análise fatorial confirmatória também foi testada, obtendo-se ajustamento do modelo, de acordo com o aceitável para os padrões de validação.

**Conclusão:**

a versão da *Family Integration Experience Scale: Chronic Illness* apresentou evidências de validação e pode ser considerada como um instrumento válido e confiável na cultura brasileira. A *Family Integration Experience Scale: Chronic Illness* , na versão português do Brasil, pode ser utilizada para medir a experiência da integração familiar na doença crônica.

## Destaques

**(1)** Validação do Family Integration Experience Scale: Chronic Illness (FIES:CI).

**(2)** Escala de experiência de integração familiar na doença crônica.

**(3)** O manejo ideal das doenças crônicas envolvendo interações com profissionais de saúde.

**(4)** Estratégias de fortalecimento das relações familiares no contexto da doença crônica.

**(5)** Processo de integração usado pelos membros da família para se adaptarem à doença.

## Introdução

 As inovações científicas e tecnológicas na atenção à saúde neonatal e pediátrica levaram a importantes mudanças na epidemiologia das doenças crônicas nessas fases do ciclo vital. As doenças infecciosas diminuíram substancialmente e a expectativa de vida aumentou, refletindo no aumento da prevalência de doenças crônicas ^(^ 1 ^)^ . 

 Na atualidade, as doenças crônicas representam a maior causa de morte e de incapacidade no mundo, pois cerca de 74% das mortes ocorridas são a elas atribuídas ^(^ 2 ^)^ . No Brasil, quanto à doença crônica na criança, dados indicam que de 9 a 11% das crianças e dos adolescentes brasileiros são portadores de doença crônica – distribuídos em 9,1% das crianças de zero a cinco anos; 9,7% daquelas de seis a treze anos e 11% dos adolescentes de catorze a dezenove anos ^(^ 3 ^)^ . 

 A *American Academy of Pediatrics* (AAP) esclarece que uma condição crônica de saúde pode durar de três meses até toda a vida, afetando o indivíduo em suas atividades diárias. Em 2020, nos Estados Unidos da América (EUA), entre 10 e 20 milhões de crianças e adolescentes sofriam pelo menos uma condição crônica, de um total de 72 milhões de norte-americanos menores de 18 anos – isso somaria uma porcentagem de crianças com doenças crônicas entre cerca de 13 e 27% ^(^ 4 ^)^ . 

 Na infância, as doenças crônicas geralmente causam sequelas e limitações ao paciente por um longo tempo, tendo grande impacto em muitos aspectos da vida da criança e de sua família ^(^ 5 ^)^ . O manejo ideal de doenças e condições crônicas de saúde envolve adaptações nas rotinas diárias das crianças e suas famílias e nas interações com profissionais e serviços de saúde. 

 À medida que as famílias tentam se adaptar ao adoecimento crônico da criança, fazem escolhas em relação às suas experiências e reconhecem sua vulnerabilidade e a atual realidade do adoecimento crônico ^(^ 6 ^-^ 7 ^)^ . Esse processo contínuo é denominado “reintegração familiar”; nele, as famílias passam a utilizar estratégias intencionais de cuidado e estabelecem um compromisso dentro da família com o objetivo de melhorar a vida familiar atual e futura ^(^ 7 ^)^ . 

 A reintegração familiar envolve processos de integração, que são usados pelos membros da família para se adaptarem à realidade e às incertezas da doença crônica, ao longo do ciclo de vida familiar no contexto da doença crônica. A reintegração familiar possibilita a capacitação da família, auxiliando-a a se adaptar à realidade e a fazer escolhas dentro da experiência da doença crônica ^(^ 6 ^-^ 7 ^)^ . Os processos de integração incluem o reconhecimento da vulnerabilidade familiar e individual dos membros da família, as estratégias intencionais de cuidado familiar, como planejar o monitoramento e a proteção, e o engajamento familiar com a doença crônica: como se conectar, ponderar, relacionar e lutar ^(^ 7 ^)^ . 

 O apoio mútuo entre os familiares se torna um facilitador para o processo de adaptação e para os cuidados de crianças com doenças crônicas. Famílias que compartilham as decisões e os cuidados da criança doente têm menos dificuldades na vida familiar e no controle da doença, assim como menor vulnerabilidade social ^(^ 8 ^-^ 10 ^)^ . Para os pais de criança com condição crônica, é fundamental o apoio emocional entre os parceiros – da família, de amigos e de profissionais de saúde – para lidar com a condição do filho ^(^ 11 ^)^ . 

 Enfermeiros são indispensáveis para auxiliar essas famílias a encontrar a melhor forma de integração. Diante de uma situação de doença, cada família agirá de uma maneira diferente e isso dependerá das suas experiências prévias, das suas crenças, dos valores de cada membro familiar, além da influência e do espaço que desenvolvem e ocupam na sua rede organizacional ^(^ 12 ^)^ . Nessa experiência, membros da família interagem entre si e são influenciados uns pelos outros, auxiliando no desenvolvimento, no enfrentamento, na manutenção da integridade e na construção da saúde familiar ^(^ 7 ^,^ 13 ^-^ 14 ^)^ . 

 Os profissionais de saúde precisam conhecer essas experiências de integração familiar no contexto da doença crônica, para que possam elaborar abordagens centradas na família ^(^ 5 ^-^ 7 ^,^ 13 ^-^ 14 ^)^ . Para o atendimento, é necessário compreender as experiências de integração familiar nas diversas condições crônicas de saúde. No entanto, a identificação dos processos de integração familiar na realidade brasileira é limitada pela falta de medidas válidas e confiáveis que capturem essas experiências. 

 A *Family Integration Experience Scale: Chronic Illness* (FIES:CI) é uma escala criada em 2020, por enfermeiras norte-americanas, a partir do modelo teórico *Reintegration Within* *Families in the Context of Chronic Illness Model* (RFCCI), com o objetivo de descrever os processos familiares utilizados para manejar a vida familiar no contexto de uma doença crônica ^(^ 6 ^)^ . 

 O instrumento aborda alguns conceitos centrais, como: vulnerabilidade familiar, estratégias de cuidado familiar, ponderação familiar, relacionamento familiar, mudança familiar, conexão familiar e luta familiar ^(^ 6 ^-^ 7 ^)^ . É composto por 9 itens, apresentados em formato analógico-visual, para capturar o subjetivo de cada processo e aumentar a capacidade de medida ^(^ 6 ^)^ . 

 A escala foi testada no contexto da cultura norte-americana e mostrou-se confiável e aplicável ^(^ 6 ^)^ . Acredita-se que a FIES:CI será aplicável e útil para a enfermagem pediátrica brasileira, por proporcionar maior compreensão da integração e da vulnerabilidade familiar diante da doença crônica, possibilitando propor estratégias de cuidado e fortalecimento das relações familiares no contexto da doença crônica da criança. 

Na realidade brasileira, existe uma lacuna no que diz respeito a instrumentos ou escalas que descrevam processos familiares. Por se tratar de uma escala de fácil compreensão e autoaplicável, a versão brasileira da FIES:CI poderá ser utilizada, pela enfermagem pediátrica, para conhecer as famílias de crianças com condições crônicas de saúde e para permitir a proposta de estratégias para auxiliar essas famílias no dia a dia com suas crianças.

 O objetivo deste estudo foi realizar a adaptação transcultural e a validação psicométrica da *Family Integration Experience Scale: Chronic Illness* (FIES:CI) para a língua portuguesa falada no Brasil. 

## Método

### Delineamento do estudo

 Trata-se de estudo psicométrico de Adaptação Transcultural (ATC) e validação do instrumento FIES:CI para a língua portuguesa falada no Brasil. Para tanto, foram seguidos os procedimentos recomendados na literatura ^(^ 15 ^)^ . Este estudo foi organizado de acordo com as recomendações do guia *Revised Standards for Quality Improvement Reporting Excellence* (SQUIRE 2.0), da Rede Equator. 

### Cenário

O estudo foi dividido em duas fases, sendo a primeira a realização da ATC da escala FIES:CI para a língua portuguesa falada no Brasil, contemplando as etapas: tradução; consenso das traduções; retrotradução; comitê de especialistas; revisão de pré-finalização; testagem cognitiva e validação linguística e análise dos comentários.

Foi realizada a tradução por dois tradutores fluentes no idioma inglês – idioma da versão original. Uma tradução foi feita por profissional da área de saúde que conhecia os objetivos do estudo e a outra, por professor de inglês que não conhecia os objetivos do estudo (tradução cega), gerando assim duas versões. Após, realizou-se uma conciliação das duas versões obtidas da etapa inicial de tradução, elaborando assim a primeira versão da escala.

 Posteriormente, a primeira versão conciliada foi enviada para um tradutor nativo na língua inglesa, que realizou a retrotradução, isto é, a volta do texto para o idioma de origem ^(^ 15 ^-^ 16 ^)^ . Essa mesma versão conciliada foi enviada para o comitê de especialistas, composto por profissionais de saúde – brasileiros e bilíngues – com formação e/ou experiência em cuidado familiar, psicometria e linguística. O papel do comitê de especialistas foi consolidar a versão final do instrumento. 

Os especialistas avaliaram a versão traduzida quanto à análise das equivalências conceitual, cultural, semântica e idiomática. Também se solicitou aos especialistas a análise de itens com objetivo da validação do conteúdo do instrumento. O contato com os especialistas aconteceu por meio de correio eletrônico.

Na segunda fase do estudo, foi realizada a verificação de evidências de validade e confiabilidade da versão brasileira da escala, por meio da validação de construto. O instrumento foi aplicado inicialmente em cinco familiares de crianças ou adolescentes com doença crônica, para a realização do pré-teste. Posteriormente, realizaram-se a testagem cognitiva e a validação linguística. Após a aplicação do pré-teste, foi definida a versão final do instrumento.

### Participantes

 A avaliação das propriedades psicométricas da escala foi realizada mediante estudo transversal, com 230 familiares de crianças e adolescentes, com até 19 anos e com doenças crônicas, que estavam em acompanhamento no ambulatório de um hospital-escola público na cidade de São Paulo. A determinação da amostra foi por conveniência e superou o recomendado pela literatura (de 10:1), possibilitando análises mais precisas ^(^ 17 ^-^ 18 ^)^ . O familiar respondente da pesquisa deveria ter mais de 18 anos e morar na mesma residência que a criança ou o adolescente. 

### Período

A coleta foi realizada entre os meses de março e maio de 2023.

### Instrumento

 A FIES:CI é uma escala autoaplicável e inicia-se com um parágrafo introdutório instrucional, no qual o membro da família é instruído a marcar o ponto apropriado ao longo da linha analógica-visual que melhor descreva as experiências dos membros da família de integração da doença ou condição crônica na vida familiar. Em seguida, os nove itens são apresentados, no formato de pontuação da escala analógica-visual de 0 a 100. Três itens são pontuados inversamente para análise: cuidado familiar, crescimento familiar e importância do relacionamento familiar. A soma dos escores pode completar de 0 a 900, sendo que escores mais baixos indicam maior integração da doença crônica na vida familiar ^(^ 6 ^)^ . 

### Coleta dos dados

Os participantes foram selecionados durante o período de espera da consulta ambulatorial, sendo convidados a responder a um questionário de caracterização demográfica e à versão final do FIES:CI.

O preenchimento do instrumento levou cerca de 10 minutos e foi realizado no ambulatório, antes ou após a consulta da criança/do adolescente. Dados complementares, como diagnóstico e tempo de diagnóstico, foram coletados no prontuário do paciente.

### Análise dos dados

 A validade de construto é a extensão em que um conjunto de variáveis realmente representa o construto a ser medido. No presente caso, ela foi testada com Análise Fatorial Confirmatória (AFC), utilizando-se o Método da Máxima Verossimilhança e utilizando o Pacote estatístico R, versão 4.3.0. Foram aceitáveis itens com carga fatorial maior do que 0,30 ^(^ 17 ^,^ 19 ^)^ . 

 Para os índices de ajustamento na AFC, foram testados: *Comparative Fit Index* (CFI) >0,90; *Tucker-Lewis Index* (TLI) >0,90; *Root Mean Square Error of Aproximation* (RMSEA) <0,08) e *Root Mean Square of Residuals* (RMSR) <0,08 ^(^ 17 ^,^ 19 ^-^ 20 ^)^ . Foi calculado o R-quadrado, que determina a proporção de variância na variável dependente que pode ser explicada pela variável independente; ele representa a proporção da variabilidade na variável resposta explicada pela variável preditora ou pela variável explanatória. Conhecido como coeficiente de determinação, ele dá a ideia de quão bem os dados se ajustam no modelo ^(^ 21 ^)^ . 

 A confiabilidade da escala foi testada por meio da análise da consistência interna, utilizando os coeficientes de alfa de Cronbach (α) e Ômega de McDonald (ωt), avaliando até que ponto as partes da escala medem a mesma característica – se seus índices valem até um. Quanto mais alta for a confiabilidade, mais exata é a consistência interna medida. Para este estudo, adotou-se um coeficiente de 0,7 como satisfatório – medida aceita e considerada internacionalmente ^(^ 17 ^)^ . 

 A estabilidade ou reprodutibilidade do instrumento (teste-reteste) foi analisada pelo Coeficiente de Correlação Intraclasse (ICC) ^(^ 17 ^,^ 22 ^)^ . 

 Os dados foram analisados por meio do pacote estatístico R, versão 4.3.0 *.* Utilizou-se a estatística descritiva, tanto para caracterização da amostra quanto para o cálculo dos valores de frequência, proporções, médias, desvio-padrão e valores mínimos e máximos. O nível de significância adotado nos testes foi de 5%. 

### Aspectos éticos

Após a autorização das autoras da escala original, o projeto foi submetido ao Comitê de Ética e Pesquisa da Escola de Enfermagem da Universidade de São Paulo, obtendo o Parecer nº 5.592.795. Após esclarecimentos sobre o objetivo e os procedimentos do estudo, os participantes assinaram o Termo de Consentimento Livre e Esclarecido.

## Resultados

 A Etapa 1, considerada como o processo de adaptação do FIES:CI, compreendeu as traduções, a síntese das traduções e a retrotradução, conforme apresentado na Figura 1 . Não havendo grandes divergências nas duas versões retrotraduzidas, foi elaborada a Versão Português 1 (VP1). A VP1 foi encaminhada para a avaliação do comitê de especialistas, com poucas sugestões de alterações, conforme demonstrado na Figura 2 , obtendo 80% de concordância entre os especialistas – construindo-se a Versão Português 2 (VP2). 

Na Etapa 2, participaram do estudo 230 familiares de pacientes de 1 a 19 anos, com condições crônicas de saúde, incluindo mãe (78,2%), pai (18,70%), avó (2,17%) e tia (0,87%). Em relação à escolaridade: 21,30% concluíram o nível superior; 1,74% tinham a formação técnica; 56,09% tinham o ensino médio completo e 20,87% haviam concluído o ensino fundamental.

Em relação à renda dessas famílias, 51,53% consideram a renda familiar suficiente, 45,85% consideram ter renda insuficiente e 2,62% relataram sobrar dinheiro no final do mês. Ao serem questionados em relação à renda antes da doença da criança, 65,7% dos familiares consideravam que a renda familiar era suficiente; 19,65% consideravam a renda insuficiente e 15,28% relataram que sobrava dinheiro no final do mês.

Das famílias participantes, 162 tinham outros filhos e 68 famílias tinham somente um filho: a criança participante do estudo. A quantidade de pessoas que moravam na mesma residência variou de uma a oito pessoas.

Quanto às crianças e aos adolescentes, 132 (57,3%) eram do sexo masculino e 98 (42,6%) do sexo feminino. A idade variou de um a 19 anos.

Os diagnósticos das crianças foram distribuídos pelos sistemas, sendo divididos em doenças neurológicas (9,3); doenças endócrinas; doenças renais; doenças respiratórias (13,4%); doenças de pele (3,9%); doenças hepáticas (4,3%); doenças do sistema digestório (10%); doenças do sistema geniturinário (2,61%); doenças de sistema imunológico (6,09%); doenças cardíacas (3,04%); doenças genéticas (6,9); câncer (3,4%) e síndromes raras (14,3%).

**Figura 1 -:** Etapas do processo de adaptação transcultural da FIES:CI. São Paulo, SP, Brasil, 2023

*VP1=Versão Português 1

^†^ VP2=Versão Português 2

**Figura 2 -:** Itens alterados após a avaliação do comitê de especialistas. São Paulo, SP, Brasil, 2023

Como se trata de um modelo bidimensional, a escala foi testada em seus dois fatores: Fator 1, que compreende os itens Q1, Q2, Q5, Q7 e Q9, sendo considerados fatores negativos; e Fator 2, com os itens: Q3, Q4, Q6 e Q8, considerados fatores positivos.

Realizou-se a análise descritiva do desempenho dos itens da escala. O alfa de Cronbach foi usado para medir a confiabilidade interna, sendo igual a 0,81, podendo-se afirmar que 81% da variabilidade do fenômeno podem ser explicados por essa escala. Na análise do alfa, nos diferentes fatores da escala, a confiabilidade do Fator 1 foi de 0,81, enquanto no Fator 2 foi de 0,60.

Realizou-se também, como teste de confiabilidade, o ômega de McDonald, obtendo-se um coeficiente de 0,81 para a consistência interna da escala, apresentando um ômega de maior valor no Fator 1 (0,81) e no Fator 2 (0,61).

 A caracterização de cada item da escala, individualmente, foi testada e demonstrou que a exclusão de itens com baixo valor de confiabilidade interna não melhoraria o valor do alfa de Cronbach nos fatores, conforme apresentado na Tabela 1 . 

 A correlação entre os dois fatores da escala foi de -0,637, com intervalo de confiança de 95% -0,864 a-0,409, conforme apresentado na Tabela 2 . 

O modelo ajustado apresentou adequados índices de qualidade de ajustamento entre os itens da escala, no entanto, a correlação entre os seus dois fatores foi negativa (-0,637). Fato esperado, devido à inversão entre os fatores da escala.

 A RMSEA foi de 0,068 (IC90%: 0,044; 0,091), valor adequado e indicativo de ajuste do modelo à estrutura fatorial. O valor do *Standardized Root Mean Square Residuals* (SRMR) foi de 0,067 – também demonstrando ajuste do modelo à estrutura fatorial. As medidas de ajuste incrementais foram TLI de 0,89 e CFI de 0,92. Assim, a estrutura do instrumento se mostrou adequada para avaliação do construto investigado. 

Para avaliar a confiabilidade da escala, foi realizado também o teste-reteste. Após quatro semanas, a escala foi reaplicada a 35 familiares, obtendo-se um valor de ICC de 0,998 – demostrando forte correlação.

**Tabela 1 -:** Estatística descritiva dos itens que compõem a escala. São Paulo, SP, Brasil, 2023

**Fator**	**Item**	n*	**Mín** ^†^	**Máx** ^‡^	**Média**	**Desvio-Padrão**	**CI** ^§^	**Alfa de Cronbach Cdsi** ^||^	Ômega de McDonald **Cdsi** ^||^
**f** 1 ^¶^	1	230	0	100	48,63	39,52	0,582	0,781	0,782
	2	230	0	100	28,30	35,89	0,592	0,778	0,780
	5	230	0	100	31,16	37,29	0,613	0,772	0,773
	7	230	0	100	47,15	40,86	0,595	0,777	0,778
	9	230	0	100	38,30	41,21	0,621	0,769	0,769
**f** 2**	3	230	0	100	80,30	30,21	0,349	0,557	0,560
	4	230	0	100	82,44	26,74	0,501	0,446	0,469
	6	230	0	100	84,15	29,21	0,304	0,590	0,595
	8	230	0	100	82,47	29,29	0,389	0,525	0,580

* n = Número absoluto

† Mín = Mínimo

‡ Max = Máximo

§ CI = Correlação Item-Total

|| Cdsi = Coeficiente do domínio sem o item

¶ f1 = Fator 1

** f2 = Fator 2

**Tabela 2:** - Distribuição das cargas fatoriais da escala. São Paulo, SP, Brasil, 2023

Fator	Item	Carga fatorial	Intervalo de confiança	R-quadrado
**f** 1*	1	0,644	0,537 a 0,751	0,416
** **	2	0,715	0,575 a 0,854	0,513
** **	5	0,698	0,578 a 0,818	0,489
** **	7	0,653	0,544 a 0,763	0,429
** **	9	0,684	0,570 a 0,798	0,470
**f** 2 ^†^	3	0,654	0,424 a 0,883	0,429
	4	0,633	0,385 a 0,882	0,403
	6	0,350	0,103 a 0,596	0,123
** **	8	0,425	0,184 a 0,666	0,181

* f1 = Fator 1

† f2 = Fator 2

## Discussão

A presente pesquisa teve como objetivos realizar a adaptação transcultural e validar a FIES:CI para a língua portuguesa falada no Brasil. A FIESC:CI é uma escala que se propõe a descrever a experiência da integração familiar no contexto da doença crônica.

 Os resultados deste estudo garantem a validade e a confiabilidade da versão brasileira da FIES:CI. Este é o primeiro instrumento brasileiro que visa medir a experiência de integração familiar no contexto da doença crônica. Um ponto forte da FIES:CI é que ela se baseia em uma estrutura conceitual sólida, construída em décadas de pesquisa, que permite avaliar e intervir na integração familiar no contexto da doença crônica ^(^ 6 ^-^ 7 ^,^ 23 ^)^ . 

 Em uma pesquisa de ATC, deve-se avaliar as equivalências semântica, linguística e contextual entre os itens originais e traduzidos; também deve ser realizada a análise das propriedades psicométricas da nova versão do instrumento. A pertinência dos conceitos e dos domínios compreendidos pelo instrumento original na nova cultura também deve ser avaliada, assim como deve-se considerar a adequação de cada item do instrumento original em termos da capacidade de representar tais conceitos e domínios na população-alvo ^(^ 24 ^)^ . 

 Na tradução e na adaptação da FIES:CI, utilizou-se um método sistemático e rigoroso – o que garantiu a qualidade da medida e forneceu evidências da validade, da aceitabilidade e da viabilidade do conteúdo da versão brasileira da escala ^(^ 15 ^-^ 18 ^,^ 23 ^-^ 25 ^)^ . Não houve grandes discrepâncias nas versões apresentadas pelos tradutores, sendo que aquelas que surgiram foram prontamente solucionadas pelas pesquisadoras principais do estudo. 

 Na avaliação das propriedades psicométricas, a escala se mostrou fácil de ser aplicada e demonstrou evidências de validade. Essas evidências foram confirmadas por meio da análise fatorial dos principais componentes da escala – os itens que compõem os fatores indicam que a variância dessas variáveis é reproduzida por fatores comuns ^(^ 19 ^-^ 20 ^)^ . 

 Verificou-se, com a análise fatorial, que todos os itens da escala têm papel importante na descrição do construto e que as cargas fatoriais da versão brasileira da FIES:CI não divergem da versão original, mantendo-se a composição da escala ^(^ 17 ^,^ 23 ^,^ 25 ^)^ . Com a análise correlacional entre os itens da escala e a escala global, concluiu-se que o instrumento pode medir a integração familiar de crianças e adolescentes com condições crônicas, pois mostra que as correlações entre todos os itens e a escala global são mais fortes do que as correlações apenas entre os itens ^(^ 17 ^,^ 19 ^)^ . 

 No instrumento original, a análise fatorial explicou 63,8% de variância, com um modelo bidimensional com dois fatores: o Fator 1, considerado o fator de integração negativa, que avalia a vulnerabilidade familiar; as mudanças da família; o sofrimento da família; a sobrecarga da família; e as preocupações financeiras; e o Fator 2, o fator positivo, que inclui estratégias de cuidado; importância das relações familiares e reflexão e crescimento da família ^(^ 6 ^)^ . 

 Apoiando a validade do construto da versão brasileira, a consistência interna e a confiabilidade teste-reteste tiveram resultados excelentes, confirmando que se trata de uma medida confiável. O valor da consistência interna conferida pelo alfa de Cronbach (0,81) superou o valor obtido no instrumento original (0,79) ^(^ 6 ^)^ . A interpretação do coeficiente alfa é geralmente preferida em detrimento de outras interpretações; no entanto, ainda não há consenso na literatura acerca da sua interpretação ^(^ 26 ^)^ . 

 Neste estudo, diferentemente do estudo original, publicado anteriormente para desenvolver e validar a escala FIES:CI ^(^ 6 ^)^ , a confiabilidade foi avaliada usando-se também o coeficiente ômega de McDonald’s, por ele fornecer uma estimativa mais restritiva da confiabilidade ^(^ 26 ^)^ . 

 Considerando a realização do teste-reteste, um estudo para adaptar e validar a *Family* *Intensive Care Units Syndrome Inventory* , para a cultura iraniana, enfatizou a importância de sua realização. Os autores reaplicaram o instrumento duas semanas após a sua primeira aplicação e o Coeficiente de Correlação Intraclasse (ICC) encontrado foi de 0,97 – valor similar ao encontrado neste estudo ^(^ 22 ^,^ 27 ^)^ . O intervalo de tempo recomendado entre as duas medidas é de quatro semanas, para evitar a variação da percepção dos sintomas positivos ou negativos e os problemas de memória ^(^ 25 ^)^ . As mudanças no estado de saúde da criança que ocorrem entre os testes também podem influenciar as respostas dos entrevistados e diminuir a confiabilidade teste-reteste. 

 As competências familiares medidas na FIES:CI – como estratégias para o cuidado, tomada de decisão e apoio financeiro – auxiliam no desenvolvimento de competências voltadas à resolução de problemas, reduzindo a chance de um desfecho negativo ^(^ 28 ^)^ . As relações familiares satisfatórias funcionam como fatores de proteção contra os comportamentos de risco para a família e seus membros ^(^ 28 ^-^ 29 ^)^ . 

 Um estudo chinês, para construção e validação de um instrumento que mede a regulação e a integração familiar, confirmou que estas são necessárias, pois crianças com famílias com excelente regulação e integração têm 3,6 vezes mais probabilidade de serem saudáveis em relação a crianças com famílias com dificuldades nesses dois domínios. A pesquisa também revelou que o conflito familiar está associado à probabilidade 38% menor de serem saudáveis ^(^ 13 ^)^ . 

 Ao validar as propriedades psicométricas do instrumento *Family Health Scale* para a versão brasileira, os autores descreveram a importância de considerar todos os fatores da escala – entre eles, medidas como funcionamento familiar, comunicação, resolução de problemas, *coping* , suporte emocional e econômico – como temas a serem abordados ao se trabalhar com a saúde da família ^(^ 28 ^)^ . Os autores ainda recomendaram que profissionais de saúde explorem em suas pesquisas questões como os vínculos de apoio intra e extrafamiliar, a fim de potencializar a compreensão sobre a saúde ^(^ 28 ^-^ 29 ^)^ . 

 Instrumentos de medida precisos, com validade e que abordem fenômenos de interesse das áreas da Enfermagem e da Saúde, são fundamentais para auxiliar na prática profissional e no desenvolvimento de pesquisas ^(^ 30 ^)^ . A FIES:CI versão brasileira foi considerada pelos participantes um instrumento de fácil compreensão – seu bom desempenho psicométrico decorre dessa facilidade de preenchimento. Para um instrumento de medida ser considerado bom, ele precisa ser bem compreendido pelos seus respondentes, ser pragmático, reduzir a sobrecarga do respondente e capturar os domínios que se propõe a medir ^(^ 31 ^)^ . 

A escala foi nomeada, em sua versão brasileira, como Escala de Experiência de Integração Familiar na Doença Crônica, buscando ser a mais próxima possível da denominação da escala original. Ela apresenta indícios de confiabilidade e validade e se encontra pronta para ser utilizada na cultura brasileira.

A aplicação do instrumento foi realizada em um ambulatório, durante a espera para o atendimento. Muitas entrevistas foram interrompidas pelas consultas médicas, precisando ser reiniciadas – o que foi um fator limitante do estudo. Houve, também, muitos estímulos externos, o que trouxe distração para alguns participantes.

Em relação à sua aplicabilidade, os participantes não consideraram o instrumento difícil de ser compreendido; no entanto, no primeiro item (“Nossa família está se sentindo vulnerável com esta doença crônica”), a palavra “vulnerabilidade” representou dificuldade de compreensão para alguns poucos participantes, sendo necessária uma explicação sobre o conceito da palavra.

A coleta de dados em apenas um local foi entendida como uma limitação, apesar de ser realizada em um serviço de referência, pois as famílias, em sua maioria, residiam na região metropolitana. Sabendo das diferenças culturais e socioeconômicas do nosso país, acredita-se na importância de a escala ser aplicada em outras regiões do Brasil.

Acredita-se, também, que essa escala poderá ser utilizada como ferramenta para avaliação da experiência da integração familiar na doença crônica, o que auxiliará profissionais de saúde a estabelecer melhor planejamento e melhor desenvolvimento de estratégias de atendimento e cuidado às famílias.

A Escala de Experiência de Integração Familiar na Doença Crônica pode apoiar a descrição e a comparação das experiências dos membros da família em várias situações de doenças crônicas. Além de medir simultaneamente as experiências, ela pode representar a adição de uma ferramenta valiosa para informar estrategicamente as ações terapêuticas de enfermagem familiar no contexto da doença crônica.

## Conclusão

A Escala de Experiência de Integração Familiar na Doença Crônica poderá contribuir como um instrumento para compreender como as famílias se integram nas diversas situações da doença crônica com as situações estressantes no seu cotidiano, conhecendo sua vulnerabilidade e como essas famílias se fortalecem diante das diversas situações da doença.

A utilização desse instrumento na avaliação familiar pode proporcionar maior interação dos profissionais de saúde com as famílias, estimulando o planejamento de intervenções específicas junto a cada família, colaborando no processo de integração familiar na adaptação à doença e incentivando as famílias a buscarem estratégias de fortalecimento das relações familiares no contexto da doença crônica da criança. Ele poderá ser usado na pesquisa, no campo da Enfermagem, possibilitando a comparação entre diversas realidades – nacionais e internacionais.

A escala foi adaptada e validada em famílias de crianças e adolescentes com doenças crônicas. Ela apresenta evidências de validade de conteúdo, construto e critério suficientes, quanto à qualidade do instrumento, para descrever a experiência da integração familiar na doença crônica da criança.

Sugere-se a ampliação da amostra para famílias de adultos e idosos com condições crônicas, verificando como o instrumento se comporta também nessas populações.
